# Identification of Pathogenic Mutations in Primary Microcephaly- (MCPH-) Related Three Genes *CENPJ*, *CASK*, and *MCPH1* in Consanguineous Pakistani Families

**DOI:** 10.1155/2022/3769948

**Published:** 2022-03-03

**Authors:** Niaz Muhammad Khan, Muhammad Shareef Masoud, Shahid Mahmood Baig, Muhammad Qasim, Junlei Chang

**Affiliations:** ^1^Department of Bioinformatics and Biotechnology, Government College University Faisalabad, Faisalabad, Pakistan; ^2^Department of Biological and Biomedical Sciences, Agha Khan University Karachi, Pakistan; ^3^Pakistan Science Foundation, 1-Constitution Avenue, G-5/2 Islamabad, Pakistan; ^4^Shenzhen Key Laboratory of Biomimetic Materials and Cellular Immunomodulation, Institute of Biomedicine and Biotechnology, Shenzhen Institute of Advanced Technology, Chinese Academy of Sciences, Shenzhen 518055, China

## Abstract

Microcephaly (MCPH) is a developmental anomaly of the brain known by reduced cerebral cortex and underdeveloped intellectual disability without additional clinical symptoms. It is a genetically and clinically heterogenous disorder. Twenty-five genes (involved in spindle positioning, Wnt signaling, centriole biogenesis, DNA repair, microtubule dynamics, cell cycle checkpoints, and transcriptional regulation) causing MCPH have been identified so far. Pakistani population has contributed in the identification of many MCPH genes. WES of three large consanguineous families revealed three pathogenic variants of *MCPH1*, *CENPJ*, and *CASK*. One novel (c.1254delT) deletion variant of *MCPH1* and one known (c.18delC) deletion variant of *CENPJ* were identified in family 1 and 2, respectively. In addition to this, we also identified a missense variant (c.1289G>A) of *CASK* in males individuals in family 3. Missense mutation in the CASK gene is frequent in the boys with intellectual disability and autistic traits which are the common features that are associated with FG Syndrome 4. The study reports novel and reported mutant alleles disrupting the working of genes vital for normal brain functioning. The findings of this study enhance our understanding about the genetic architecture of primary microcephaly in our local pedigrees and add to the allelic heterogeneity of 3 known MCPH genes. The data generated will help to develop specific strategies to reduce the high incidence rate of MCPH in Pakistani population.

## 1. Introduction

Primary microcephaly is a rare genetically heterogenous neurodevelopmental condition that specifically affects the cerebral cortex, and the affected individual has a reduced brain weight than the individuals of the same age, ethnicity, and gender [1, 2]. It is characterized by an architecturally normal brain with mild to moderate intellectual disability [3]. Clinically, the severe MCPH is defined as the occipitofrontal head circumference (OFC) of more than 3SD below the mean for gender, age, and ethnicity [4, 5].

It is a recessive hereditary disorder, and most of the MCPH causing mutation occur in genes that are essential for maintaining proper cell cycle and integrity of the cellular genome [6]. Phenotypically and genetically microcephaly is a very heterogeneous disorder with over 900 OMIM phenotype entries and at last count 25 different types of genes with diverse form of linked variants [6, 7]. Majority of these genes express during the proliferation of neural precursor cells (NPCs) in the ventricular zone of the cerebral cortex [8]. The most frequent cause of this clinical disorder is the mutation in *WDR62* (WD repeat-containing protein 62) and *ASPM* (abnormal spindle-like, microcephaly-associated) genes which together responsible for more than half (½) the cases of MCPH, followed by *MCPH1* which is the first and third most common causes of MCPH [9–11]. At cellular level, *MCPH1* encode a multifunctional protein that play an important role in chromosome condensation, DNA damage response, cell cycle control, and DNA repair [12]. Many diverse forms of this congenitally rare genetic disorder may involve a compromised division of cortical precursor cells that leads to a reduced proliferation of neurons due to imbalance among symmetric and asymmetric division of the NPCs. Ultimately, the reduced number of neurons leads to the reduced brain volume, as observed in the MCPH patients [13, 14].

Due to the common practice of consanguineous marriages in Pakistani population, the incidence rate of MCPH (though it is a rare recessive disorder) is high (1/10,000) as compared to the European white populations [15]. One reason of high prevalence of microcephaly in Pakistan is the lack of genetic counselling in the country. More than half of marriages in Pakistan are consanguineous that increases the chances of getting autosomal recessive primary microcephaly in the next generation [16]. Thus, proper screening of more population will reduce the incidence rate of MCPH in Pakistani population.

In the current study, WES and Sanger sequencing were used for the identification of genetic components involved in MCPH. Here, we report three variants in *MCPH1*, *CENPJ*, and *CASK* genes segregating with MCPH. The most frequent pathomechanism of mutant alleles in these genes is the dysfunction of MCPH proteins either through excessive apoptosis or dysregulation of cell cycle dynamics impairing mitotic neurogenesis leading to the precipitation of MCPH phenotypes [17, 18]. Findings of the current study will help to better understand the neurogenesis and pathophysiology of MCPH. Furthermore, the variants of MCPH genes reported in this study will help in devising better molecular diagnostic strategies and providing genetic counselling to the affected families.

## 2. Materials and Methods

### 2.1. Subjects and Approval of This Research Study

This study was duly approved by the Institutional Review Boards (IRBs) of Government College University Faisalabad-Pakistan and Shenzhen Institute of Advanced Technology, Chinese Academy of Sciences, China. Detailed clinical information (videos, photographs, medical records, and interviews), pedigrees, and blood samples were collected after written informed consents from the parents/guardians following the declaration of Helsinki. Patients were physically examined, and their head circumference was measured.

### 2.2. Whole-Exome Sequencing

DNA specimen from affected subjects of each pedigree were subjected to whole-exome sequencing (WES) using Illumina NovaSeq 6000 platform to recover genomic libraries and sequenced with an average of 100x coverage on an Illumina HiSeq4000 (Illumina, San Diego, CA, USA). Human reference genome sequence (GRCh37) assembly was used as reference, and reads were aligned and mapped to it. GATK version 3.7 was used for variant calling and SnpEff (version 4.2; http://snpeff.sourceforge.net/) was employed for the classification and annotation of variants. Single-nucleotide variants were filtered by using a variant quality score recalibration method. Postannotation filtration of the variants was done by using the public databases like Genome Aggregation Database (gnomAD) and 1,000 Genomes Project. All the variants with minor allele frequency (MAF) > 0.005 were discarded. Among the retained variants (MAF < 0.005), homozygous and compound heterozygous alleles were focused as the most likely transmission mode for these pedigrees was autosomal recessive. No further in silico tools were used for the deletion variants as these result in immature truncation of translation.

### 2.3. Sanger Sequencing

The predicted identified variants after whole-exome sequencing were validated through genotyping of all available family members and checked which variant is homozygously segregated in all affected individuals with the disease phenotype. Primer3 web resource was used to design a primer for Sanger sequencing (http://bioinfo.ut.ee/primer3-0.4.0/) available in Table 1. Samples were run on Applied Biosystem 3730 Genetic Analyzer using BigDye, respectively. DNASTAR (Lasergene) and Sequencher 5.4.6 (Gene Codes Corporation) were used to analyze the chromatograms.

### 2.4. In Silico Analysis of the Identified Variants

The pathogenicity of variants was ascertained according to the criteria of the American College of Medical Genetics (ACMG) (Richards et al., [19]). Bioinformatics prediction tools such as SIFT (http://sift-dna.org/sift4g), Polyphen2 Bioinformatics prediction tools such as SIFT (http://sift-dna.org/sift4g), Polyphen2 (http://genetics.bwh.harvard.edu/pph2/), Provean (WEB LINK), Fathmm (http://fathmm.biocompute.org.uk/), and CADD (https://cadd.gs.washington.edu/score) were used to predict the impacts of missense variant on protein foldings. SIFT Indel was used for predicting the effect of deletion mutation on the protein (https://sift.bii.a-star.edu.sg/www/SIFT_indels2.html).

## 3. Results and Discussion

**Figure 1:**
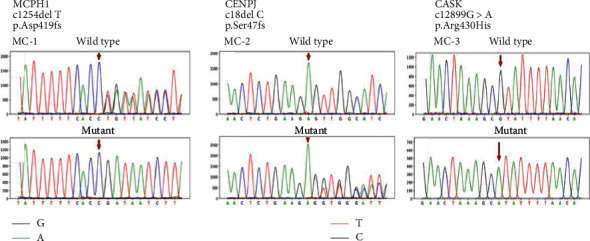
Sanger sequencing chromatograms of wild -type and mutant sequences showing deletion and substitution mutations in c.1254delT-MCPH1, c.18delC-CENPJ, and c.1289G>A-CASK.

**Figure 2:**
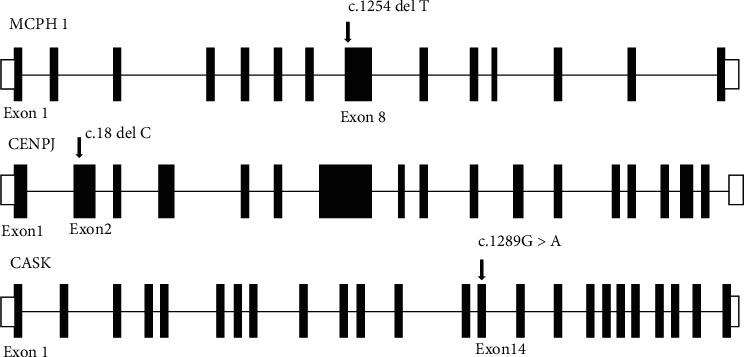
Schematic representation of exon and intronic regions of human MCPH1, CENPJ, and CASK genes along with the position of known and novel mutations. The white box represents the untranslated regions (UTR). The straight line represents the introns and the rectangle represents the exons. The asterisk sign shows the novel deletion variant in the MCPH1 gene.

We report a novel deletion variant in *MCPH1* and reported deletion and missense variants in *CENPJ* and *CASK* genes, respectively (Figure 1). Gene structures of MCPH1, CENPJ, and CASK genes are shown in Figure 2. All the affected individuals segregated MCPH in recessive inheritance pattern as their parents were normal. Detailed clinical and genetic manifestations in these three families are given in Table 2. In all the affected individuals, MCPH was present as a congenital disorder and with no history of maternal infection or head injury.

Exome sequence analysis of the proband and subsequent Sanger sequencing of all the affected members from 3 unrelated families discovered a homozygous and hemizygous variant in the three different genes, i.e., *MCPH1*, *CENPJ*, and *CASK*.

**Figure 3:**
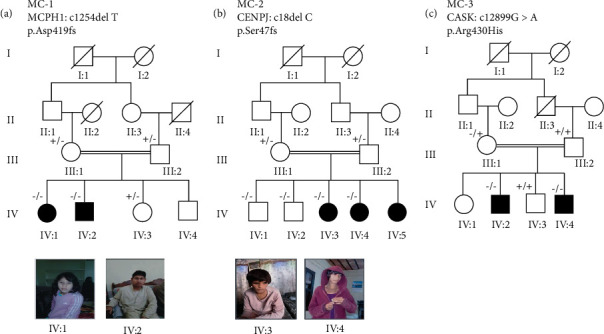
MCPH families showing autosomal and X-linked mode of disease segregation. The filled circles and squares show affected females and males. The open circles and squares show the unaffected individuals. Consanguineous marriage is represented by double lines. The (-/-) sign show the recessive homozygous individuals and (-/+). Patient's facial characteristics represented in photographs reduced head circumference with no other facial dysmorphism. Family MC-3 did not grant consent for their photographs.

In an individual (IV-1 and IV-2), from the MC-1, a novel frameshift variant c.1254delT was observed at exon 8 of the MCPH1 gene (Figure 3(a)). The Sanger sequencing demonstrates that single-base deletion c.1254delT in the *MCPH1* gene cosegregate with the disease in a homozygous manner while both parents and normal siblings of the patients were found heterozygous. This frameshift variant causes the damage to asp amino acid at position 419 and loss of function. Interestingly, in silico tool SIFT Indel predicted it pathogenic and cause nonsense-mediated mRNA decay (NMD). The literature survey of this variant showed that the variant has not been reported in the literature or found absent from the large population databases: Human genome mutation database (HGMD), gnomAD, and 1000 Genomes Project and ClinVar.

In the MC-2, three affected female individuals are presented in the fifth generation (IV-3, IV-4, and IV-5). These individuals showed typical microcephaly phenotypes which were in line with the previously reported cases. The exome sequence analysis of three patients revealed previously known homozygous frameshift variant c.18delC at exon 2 of the *CENPJ* gene [20] (Figure 3(b)). This variant causes change of serine amino acid to proline at position 7 (p. Ser7profs). Subsequent genotyping of the patient's data through Sanger sequencing confirmed it homozygous to the heterozygous carrier parents and normal siblings. This is a highly characterized pathogenic mutation for microcephaly, and further in silico analysis was not needed.

**Figure 4:**
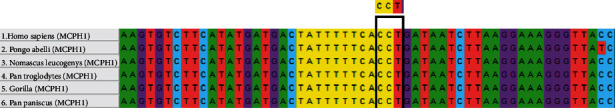
In MCPH1 gene, the novel mutation in CCT codon which is present in an evolutionary conserved region, throughout multiple species.

In MC-3, two affected male individuals IV-2 and IV-4 have symptoms of microcephaly, seizure, and epilepsy. WES and Sanger sequence analysis revealed previously reported hemizygous missense variant c.1289G>A at exon 14 of the *CASK* gene (Figure 3(c)). This mutation causes the change of arginine at position 430 to Histidine (p. Arg430His). This change is predicted highly damaging by SIFT, Polyphen2, Fathmm, and MCAP for the resulting protein (Figure 4). This mutation is present in the highly conserved region of the *CASK* protein. Screening of additional families will refine it, and such families will be of great value in defining genotype and phenotype correlations.

Mutant alleles of *MCPH1* gene have been reported with immature chromosome condensation syndrome and primary microcephaly 1. The present study reports a novel frameshift deletion mutation c.1254delT at exon 8 with modified amino acid p. Asp419fs (Figure 1). Multiple sequence alignment showed conservation of proline across species. This variant disrupted the structurally and functionally conserved domain of *MCPH1* gene resulting in disease phenotype. *MCPH1* gene plays a pivotal role in the neurogenesis of the cerebral cortex and regulation of brain size [21, 22]. In situ hybridization revealed an elevated expression of microcephalin in the fetal mouse brain especially near the lateral ventricles during neurogenesis signifying the involvement of *MCPH1* gene in the size regulation of the brain cortex [21]. The *MCPH1* mouse model revealed abnormal chromosome condensation during mitosis and a decreased skull size [23, 24].

The *CENPJ* (centromere protein j) gene having 17 exons present on chromosome 13q12.2 plays a major part in assembling, rearrangement, and integrity of the microtubules, during the neurogenesis. The structural changes or complete loss of *CENPJ* gene leads to the damaged centrosome, multiple spindle poles, cell arrest in mitosis, and loss of centrioles. The *CENPJ* protein is contained to centrosomes in interphase and to the spindle poles during mitosis [25]. Cho et al. found that the exhaustion of *CENPJ* protein impairs centrosome integrity and mitosis is arrested in cells deficient in *CENPJ* [26]. The centrosome serves as a microtubule organizing center and is crucial for the regulation of the cell division. Centrosomal mechanism is the key player in regulating brain size [27]. Interaction of *CENPJ* with other MCPH proteins like *WDR62*, *CEP152*, *STIL*, *CEP135*, and *ASPM* results in microtubules binding [12, 28], Recent studies reveal that *CENPJ* controls progenitor division and neuronal migration in the brain [29]. Until now, eight mutations are identified in this gene (including the one reported in this study) and two of them have been reported in Pakistani MCPH families [15]. MC-3 is the 6th serial Pakistani family in which this frameshift variant (c.18delC) is being reported so this *CENPJ* variant could reasonably be a founder mutation of the Pakistani population.

*CASK* (OMIM# 300172) is an X-linked gene with 27 exons which encode a protein (calcium/calmodulin-activated serine kinase), with a role in ion channel trafficking, synaptic transmembrane protein anchoring, neural development, and gene expression regulation. CASK gene expression in the mammalian brain is higher than the other organs of the body [30]. Mutations throughout this gene are known to be involved in the X-linked intellectual disabilities of varying lethality in male and female individuals [31]. The clinical symptoms associated with mutation in the *CASK* gene is gender specific. In the girls, the severe deletion mutations in the *CASK* gene is associated with ailment known as mental retardation and microcephaly with pontine and cerebellar hypoplasia (MICPCH) (OMIM#300749), while in male, boy's epileptic encephalopathies such as Ohtahara syndrome and infantile spasms are most commonly observed [32–34]. Splice site, duplication, nonsense, and deletion mutation in the *CASK* gene are less frequently observed in the male which may be due to early male lethality [31]. Missense mutation in the *CASK* gene is frequent in the boys with mental retardation and autistic traits [35]. WES and Sanger sequence analysis revealed previously reported hemizygous missense variant c.1289G>A at exon 14 of the *CASK* gene (Figure 1) [32]. This mutation causes the change of arginine at position 430 to Histidine (p. Arg430His). This change is predicted highly damaging by SIFT, Polyphen2, Fathmm, and MCAP for the resulting protein. In public databases gnomAD and ClinVar, this mutation is present with conflicting interpretation and uncertain clinical significance. Segregation analysis of the variant in the normal mother and sibling found heterozygous which is suggesting recessive carrier for this mutation. Females with missense heterozygous mutations may have very mild cognitive deficits with no microcephaly or cerebellar hypoplasia, suggesting an X-linked recessive inheritance pattern [35]. This variant is present in the highly conserved region of the *CASK* protein. Based upon the American College of Medical Genetics and Genomics guideline for variant clinical interpretation, we can confidently say that the variant c.1289G>A (p. Arg430His) is responsible for the phenotype of the patients presented in this study [36].

## 4. Conclusion

The current study reports one novel and two previously reported mutations in three Pakistani MCPH families in well-studied MCPH genes. Functional assessment of the novel mutation will clarify its impact on neurogenesis and the development of microcephaly. The findings of this study expand the mutation dataset related to MCPH in the Pakistani population and pave the way for better genotype-phenotype correlations and better understanding and management of MCPH.

## Figures and Tables

**Table 1:** Primers sequences used to amplify mutation of *MCPH1*, *CENPJ*, and *CASK.*

ID	Gene	Mutation	Forward	Reverse	Product size
MC-1	MCPH1	c.1254delT; p. Asp419fs	ACCAGGAGATCTATCATGCC	AGAAGTCACGCAACTCGAAG	375
MC-2	CENPJ	c.18delC; p. Ser7fs	GTAGCTCAATGCCCAATTGC	AGAAATGTCCACAGCTGCTC	370
MC-3	CASK	c.1289G>A; p. Arg430His	CCTGCCATAAAAATCCACTC	AGTACAGTCCCTGAAAAGCC	412

**Table 2:** Clinical and genetic manifestation of microcephaly patients.

Family individual ID	Gene	Mutation	Affected members	Age on onset (years)	Head circumference (cm)	Intellectual disability	Epilepsy	Hearing loss	Ophthalmological anomalies	Skeletal anomalies	Neurological defect
MC-1	MCPH1	c.1254delT; p. Asp419fs	2								
IV-1				8	44	+	—	+	—	—	+
IV-2				15	45	+	—	+	—	—	+

MC-2	CENPJ	c.18delC p. Ser7fs	3								
IV-3				23	37	+	+	+	—		
IV-4				17	38	+	+	+	—	—	+
IV-5				14	37	+	+	+	—	—	+

MC-3	CASK	c.1289G>A p. Arg430His	2								
IV-2				14	37	+	+	+	—	—	+
IV-4				10	43	+	+	+	—	—	+

## Data Availability

All the relevant data are included in the manuscript.
